# Cat in a Meal Tub

**Published:** 1880-05

**Authors:** 


A "Cat in a Meal Tub."—A contemporary hands us an advertisement from a city newspaper, and says: "You will see from this that certain parties contemplate a 'new departure' in medical affairs in our city. Is this another 'cat in a meal tub'?" Lecturing to females who contemplate engaging in the avocation and duties of nursing is not a new thing. It has been done in New York and elsewhere, and as to the matter of "the cat" he is just as able to judge as we are. About the inception of the late civil war we delivered a series of lectures to large numbers of ladies on the subject of nursing, and the course was repeated by invitation twice afterward during the war. Some ten or twelve years ago we made an effort to get up a training school in this city for male and female nurses, and renewed it again a few years ago; but finally failed for want of adequate co-operation and assistance. The programme proposed differed essentially from that announced in the advertisement before us, however. We do not believe the step about to be taken, if conducted in accordance with the announcement, will receive the sanction or approval of the more intelligent portion of the profession of Baltimore, or that it ought to be endorsed by any portion thereof. Nor do we believe that the laity acquainted, even in moderate degree, with the relative duties of the profession and public, will be likely to look complacently upon such wholesale and indiscriminate admission of ignorant and probably even immoral characters as will have *carte blanche*, as well as the more intelligent and moral who may be induced to the repast of the "professors," if the advertisement means what it says. Men sometimes overreach themselves in their "philanthropological" efforts before the public. Whether such has been the case in the announcement under criticism, others can judge as well as ourselves. One thing is, however, quite clear: it advertizes the four names mentioned as *unusually* charitably disposed persons.

